# Rural–urban differences in dental service use among children enrolled in a private dental insurance plan in Wisconsin: analysis of administrative data

**DOI:** 10.1186/1472-6831-12-58

**Published:** 2012-12-21

**Authors:** Pradeep Bhagavatula, Qun Xiang, Aniko Szabo, Fredrick Eichmiller, Raymond A Kuthy, Christopher E Okunseri

**Affiliations:** 1 Department of Clinical Services, Marquette University School of Dentistry, P.O. Box 1881, Milwaukee, WI, 53201-1881, USA; 2 Division of Biostatistics, Institute for Health and Society, Medical College of Wisconsin, Milwaukee, USA; 3 Delta Dental of Wisconsin, Milwaukee, USA; 4 Department of Preventive and Community, Dentistry University of Iowa College of Dentistry, Milwaukee, USA

**Keywords:** Oral health, Urban, Rural, Dental care for children, Dental Insurance, Health services accessibility

## Abstract

**Background:**

Studies on rural–urban differences in dental care have primarily focused on differences in utilization rates and preventive dental services. Little is known about rural–urban differences in the use of wider range of dental procedures. This study examined patterns of preventive, restorative, endodontic, and extraction procedures provided to children enrolled in Delta Dental of Wisconsin (DDWI).

**Methods:**

We analyzed DDWI enrollment and claims data for children aged 0-18 years from 2002 to 2008. We modified and used a rural and urban classification based on ZIP codes developed by the Wisconsin Area Health Education Center (AHEC). We categorized the ZIP codes into 6 AHEC categories (3 rural and 3 urban). Descriptive and multivariable analysis using generalized linear mixed models (GLMM) were used to examine the patterns of dental procedures provided to children. Tukey-Kramer adjustment was used to control for multiple comparisons.

**Results:**

Approximately, 50%, 67% and 68 % of enrollees in inner-city Milwaukee, Rural 1 (less than 2500 people), and suburban-Milwaukee had at least one annual dental visit, respectively. Children in inner city-Milwaukee had the lowest utilization rates for all procedures examined, except for endodontic procedures. Compared to children from inner-city Milwaukee, children in other locations had significantly more preventive procedures. Children in Rural 1-ZIP codes had more restorative, endodontic and extraction procedures, compared to children from all other regions.

**Conclusions:**

We found significant geographic variation in dental procedures received by children enrolled in DDWI.

## Background

People living in rural and inner-city regions often face significant barriers (such as lack of dental insurance, transportation and shortage of providers) in accessing dental care [1-5], have greater unmet dental need and consequently report poorer oral health status [5-7]. Studies on differences in dental service use in rural and urban areas have primarily focused on enrollees of public insurance programs and on differences in utilization rates, usually defined as the percentage of population having an annual dental visit [3-5,8]. These studies have reported profound disparities in utilization rates with residents of rural areas having significantly fewer dental visits [3-5,8].

Differences in provision of dental procedures can be used as measures of disparities in dental care. However, very few studies have examined rural vs. urban differences in provision of dental procedures [9-11]. Brennan et al., found that among Australian adults served by public dental programs, those in non-urban areas were less likely to have preventive services and more likely to receive restorative, oral surgical and prosthodontic services [9,10]. In addition, studies on variation in receipt of dental procedures among children have examined racial differences [12,13], and information on geographic variation in the use of a wider range of dental procedures is nonexistent, especially among the privately insured. This information is important for program planning and policy development aimed at reducing or eliminating differences in dental care if they exist.

Enrollees of private dental insurance plans have higher utilization rates than the uninsured or publicly insured populations [14-16], indicating better access to dental care. Private insurance enrollees have equal access to dental care; however, minorities and people living in low income areas tend to have fewer dental visits [12,13]. The association of risk factors in the receipt of dental procedures among children enrolled in private insurance and living in small rural areas and inner-city neighborhoods has not been explored. Identifying these factors is an important step in improving oral health for all enrollees, given that 50 percent of children in the United States are enrolled in private dental insurance plans [14].

In this study we examined the impact of some of the Predisposing, Enabling and Need factors (PEN factors), as described by Andersen et al. [17], on patterns of different dental procedures provided to children living in areas with different levels of urbanization in Wisconsin after adjusting for available covariates such as age and area poverty levels.

## Methods

### Data source

The subjects were enrollees of Delta Dental of Wisconsin (DDWI), the largest private insurance dental benefits plan with more than 1.25 million enrollees (~21% of people in Wisconsin) [18]. About 90 percent of Wisconsin's dentists are registered providers of DDWI’s network. DDWI provides large and small group plans through employers, as well as individual dental plans. It uses network discounted fee schedules for reimbursement of dentists [18].

### Measures

We analyzed enrollment and claims for 0-18 year old children enrolled in DDWI from 2002 to 2008. The enrollment files had information on the number of children insured for each year by ZIP code, gender, and age. The claims dataset had information on age, ZIP code of residence of the child, date of treatment delivery, and procedure code for the treatment provided. We categorized the children into one of five age groups; 0-3 years, 4-6 years, 7-9 years, 10-14 years, and 15-18 years to have a balance in the number of groups as well as the number of enrollees per group, across the various geographic regions. Child age was defined based on the child’s age at the last dental visit during the year.

We modified and used a Rural–urban Classification developed by Wisconsin Area Health Education Center (AHEC) to categorize children into six groups (AHEC categories) based on their ZIP code of residence (Table 1) [19], Personal communication from Ms. Nancy Sugden, Wisconsin AHEC]. ZIP codes within rural areas with no population center greater than 2,500 were designated as Rural 1 regions. The ZIP codes within population clusters of 2500-9999 and 10,000-49,999 people were designated as Rural 2 and Rural 3 regions, respectively. Any ZIP codes in areas with population nucleus of 50,000 up to 1 million were designated as ‘Urban’. Finally, we modified the classification by adding an additional category to the classification. We categorized ZIP codes in Greater Milwaukee Area (population over 1 million), into inner-city (ICM) and suburban-Milwaukee (SM) groups.

**Table 1:** Classification of ZIP codes based on the number of people living in a population cluster+

**AHEC Category**	**Description**
Rural 1	ZIP codes with population of **less than 2500** living in population clusters
Rural 2	ZIP codes with population of **2,500-9,999** living in population clusters
Rural 3	ZIP codes with population of **10,000 – 49,999** living in population clusters
Urban	ZIP codes with population of 50,000 up to 1 million living inside **urbanized areas outside the Milwaukee** metropolitan area
Suburban Milwaukee	Milwaukee Metropolitan area; **excluding inner-city Milwaukee ZIP codes**
Urban Milwaukee	Milwaukee county- **Inner-city Milwaukee ZIP codes**

+ Personal Communication from Ms. Nancy Sugden Director, Wisconsin Area Health Education Center.

Dental treatment procedures were identified based on Current Dental Terminology (CDT) codes and grouped into six categories. All the diagnostic CDT codes (D0100 to D0999) were categorized as such. CDT codes for oral prophylaxis (D1120), fluoride varnish (D1206), fluoride gel applications (D1203), and dental sealant placement (D1351) were categorized as preventive procedures. Restorative procedures were divided into two categories: simple (D2000 to D2430) and complex restorative procedures (D2510 to D2999). Endodontic procedures included D3000-D3999 and extraction/surgical procedure included codes for extraction of deciduous teeth (D7111), extraction of erupted teeth (D7140) and extraction of erupted teeth requiring elevation of mucoperiosteal flap (D7210).

### Conceptual model

The conceptual framework for this study is closely related to the health behavioral model proposed by Andersen et al, which describes societal and individual determinants of medical care utilization [17]. Under the individual determinants, this model proposes three sets of factors that determine the utilization of healthcare services by individuals. a) predisposing factors which are those that exist prior to disease, and can be either mutable or immutable (b) enabling factors include resources that affect one’s ability to access the health care system, and (c) need factors that reflect an illness that requires the use of services. Some of the study variables we used in this study such as; age, rurality or urbanicity place of residence and neighborhood poverty levels are examples of predisposing factors, having private dental insurance, which can also be a proxy for family socioeconomic status, is an enabling factor and the type of treatment procedures (e.g., a sealant as opposed to an endodontic procedure) received can be a proxy measure for need.

### Statistical analysis

Descriptive statistics were performed to provide estimates for the number of procedures of each type provided to children across various AHEC categories, and preventive procedures for various age groups. The claims data was aggregated to obtain the number of procedures of each type performed during a calendar year for each enrollee to obtain the averages per year per enrollee. We also calculated the average number of procedures per child per year within each ZIP code based on log transformed ZIP code poverty level to explore the relationship between area poverty and dental procedures. The information on ZIP code poverty levels was obtained from the 2000 US Census information [20]. The overall utilization rates were calculated based on the proportion of enrollees who had at least one dental visit in a given year. Utilization of a specific dental procedure was defined as the presence of a claim for such procedure and utilization rates for treatment procedures were calculated based on the number of enrollees who had at least one procedure in a year.

Multivariable analysis based on Poisson regression with random ZIP code effect was used to test for differences in the number of dental procedures of each type provided to children across the AHEC categories. The covariates in the analysis were age at time of treatment, year of treatment and log transformed poverty levels in the ZIP code. Enrollees from inner-city Milwaukee were used as the reference population for calculating the Rate ratios for provision of dental procedures. The reference group was selected based on initial analysis which showed that they were the group with lowest utilization rates. We also performed pairwise comparisons of average number of procedures between each of the AHEC categories. Tukey-Kramer adjustment was used to control for multiple comparisons. All analyses were performed using SAS version 9.2 (SAS Institute Inc Cary, NC), with PROC GLIMMIX used for the main analysis. A statistical significance level (alpha) of 0.05 was used throughout. This study was approved by the Marquette University’s Institutional Review Board.

## Results

**Figure 1:**
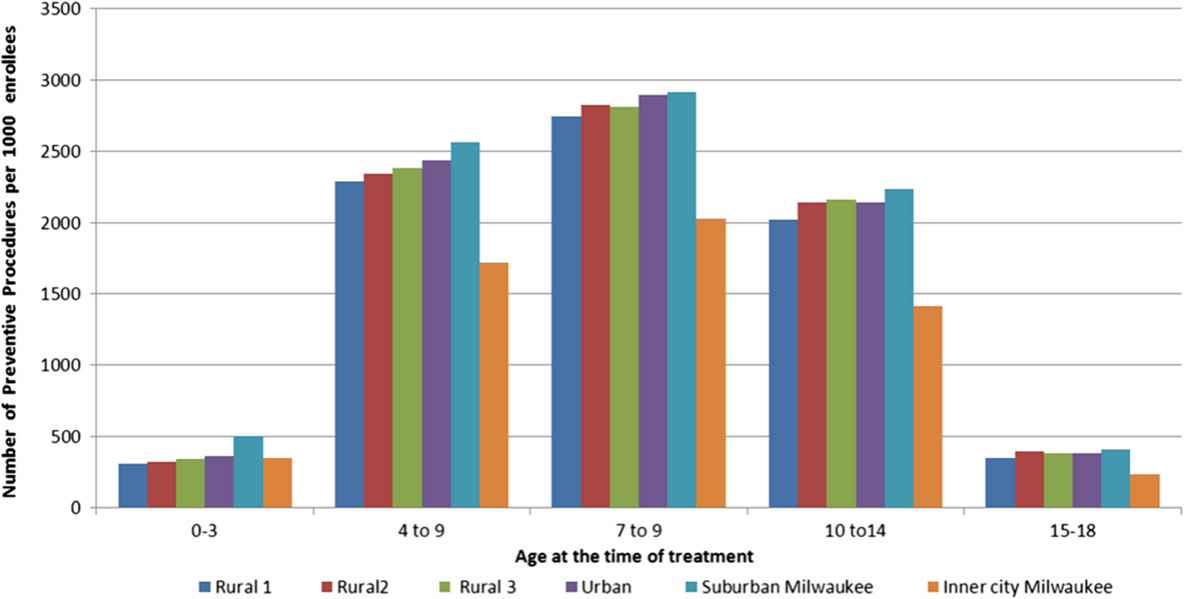
Average Number of Preventive Procedures per 1000 Children Enrolled in DDWI per year (2002-2008).

The total enrollment during the study period was 1,876,314 person-years. Table 2 displays the age and gender distribution of enrollees in DDWI during the seven years (2002 – 2008). The proportions for the different age groups and gender in all the AHEC categories included in the study were almost equal. The Urban group had the highest and inner-city Milwaukee group had the lowest number of enrollees. The average number of preventive procedures provided per 1000 enrollees is illustrated in Figure 1. The children living in inner-city Milwaukee received the fewest, and those from suburban Milwaukee received the highest number of preventive procedures for most age groups, respectively. Children in 0-3 and 15-18 age categories from all the AHEC categories received considerably fewer preventive procedures than children in other age groups.

**Table 2:** Characteristics of study population (0-18 year old children enrolled in DDWI from 2002-2008)

	**Rural 1**	**Rural 2**	**Rural 3**	**Urban**	**Suburban Milwaukee**	**Inner city Milwaukee**
**Gender**						
**Female**	162,429	129,006	87,138	336,610	137,706	48,891
	(48.37%)	(48.16%)	(48.60%)	(47.50%)	(48.62%)	(48.14%)
**Male**	169,246	136,030	90,490	356,497	141,696	51,452
	(50.40%)	(50.78%)	(50.47%)	(50.31%)	(50.03%)	(50.67%)
**Gender Unknown**	4,099	2,830	1,677	15,492	3,817	1,208
	(1.22%)	(1.06%)	(0.94%)	(2.19%)	(1.35%)	(1.19%%)
**Age**						
**0-3**	58,138	46,602	31,523	127,904	51,226	17,540
	(17.31%)	(17.40%)	(17.58%)	(18.05%)	(18.09%%)	(17.27 %)
**4-6**	45,836	36,759	24,725	99,211	41,896	14,371
	(13.65%)	(13.72%)	(13.79%)	(14.00%)	(14.79%)	(14.15%)
**7-9**	50,522	41,406	27,142	108,701	45,355	16,005
	(15.05%)	(15.46%%)	(15.14%)	(15.34%)	(16.01%)	(15.76%%)
**10-14**	98,850	79,119	52,561	206,399	81,356	30,031
	(29.44%%)	(29.54%)	(29.31%)	(29.13%)	(28.73%)	(29.57%)
**15-18**	82,428	63,980	43,354	166,384	63,386	23,604
	(24.55%)	(23.89%)	(24.18%)	(23.48%%)	(22.38%)	(23.24%)
**Total**	**335,774**	**267,866**	**179,305**	**708,599**	**283,219**	**101,551**

Numbers in parenthesis represent percentage of the total population.

Table 3 reports the utilization rates, and the average number of procedures of each type provided per 1000 enrollees per year for each of the AHEC categories during the study period. Children living in inner-city Milwaukee had fewer dental procedures for most treatment categories included in the analysis except complex restorations and endodontic procedures. Children living in Rural 1 areas received the highest number of corrective procedures (combined restorative, endodontic and extraction), and children from suburban Milwaukee received higher number of preventive procedures.

**Table 3:** Number of dental procedures per 1000 enrollees in a year and utilization rates for dental procedures among children enrolled in DDWI

	**Rural 1**		**Rural 2**		**Rural 3**		**Urban**		**Suburban Milwaukee**		**Inner city Milwaukee**	
	**No. of Proc.**	**Util. Rate**	**No. of Proc.**	**Util. Rate**	**No. of Proc.**	**Util. Rate**	**No. of Proc.**	**Util. Rate**	**No. of Proc.**	**Util. Rate**	**No. of Proc.**	**Util. Rate**
**Diagnostic**	1653	64.0%	1703	66.0%	1666	66.0%	1750	67.0%	1749	67.0%%	1314	48.0%
**Preventive**	1477	47.0%	1556	49.0%	1554	48.0%	1599	50.0%	1694	51.0%	1109	35.0%
**Simple Restorative**	558	21.0%	511	20.0%%	503	20.0%	442	18.0%	421	17.0%	362	14.0%
**Complex Restorative**	35	2.0%	31	2.0%%	28	2.0%	30	2.0%	26	1.0%	29	2.0%
**Endodontic**	31	2.0%	28	1.0%	28	1.0%	28	1.0%	23	1.0%	29	2.0%
**Extractions**	82	4.0%	78	4.0%	75	3.0%	84	4.0%	73	3.0%	61	3.0%
**Corrective (Restorative + Endodontic + Extractions)**	706	--	648	--	634	--	584	--	543	--	481	--
**Total/Overall Utilization**	3866	67.0%	3907	68.0%%	3854	68.0%	3933	69.0%	3986	68.0%	2904	50.0%

No. of Proc- Number of procedures per 1000 years of enrollment; Util. Rate- Utilization rate for a given procedure i.e. proportion of children receiving at least one procedure in an year; Total/Overall Utilization: proportion of children with at least one annual dental visit.

The proportion of children receiving any preventive procedure increased with the size of the population cluster except for inner-city Milwaukee. Children living in inner-city Milwaukee (35%), followed by children living in Rural 1 areas (47%), had the lowest utilization rates for preventive dental procedures. Children living in suburban Milwaukee had the highest utilization rates for preventive services (51%). A similar pattern was identified for diagnostic procedures where children from inner-city Milwaukee (48%), followed by those living in Rural 1 areas had lower rates (64%) and children living in suburban Milwaukee (67%) had higher rates. Children living in Rural 1 areas had the highest utilization rates for restorative and endodontic procedures of all the groups. Table 3 also shows overall dental utilization rates for enrollees from each of the ZIP code groups. Fifty percent of inner-city Milwaukee enrollees had at least one dental visit. The remaining groups had similar utilization rates (67-69%).

Rate ratios from multivariable analysis for comparing dental procedures provided to children across the AHEC categories are summarized in Table 4. Children living in Inner-city Milwaukee were used as the reference group. Compared to children in the reference group, children living in suburban Milwaukee had the highest number of preventive or diagnostic procedure and the fewest number of corrective procedures. Children from Rural 1 areas had the highest number of corrective procedures, with rate ratios of 1.71 and 2.13 for simple and complex restorations and 1.90 and 1.75 for endodontic and extraction procedures, compared to children from inner-city Milwaukee. The rates ratios for complex restorative and endodontic procedures for other AHEC groups were not significantly different when compared to the reference population.

**Table 4:** Results from multivariable analysis examining geographic variation in dental procedures among 0-18 year old children enrolled in DDWI from 2002-2008 and the impact of ZIP code level poverty

	**Ref. Group**^**$**^	**Impact of Poverty**^**+**^	**Impact of Poverty**^**+**^	**Impact of Poverty**^**+**^	**Impact of Poverty**^**+**^	**Impact of Poverty**^**+**^	**Impact of Poverty**^**+**^
	**Inner-city Milwaukee**	**Rural 1**	**Rural 2**	**Rural 3**	**Urban**	**Suburban Milwaukee**	
**Diagnostic**	1.00 a	1.20 (1.12-1.29) b	1.26 (1.17-1.36) bc	1.27 (1.16-1.38) bc	1.26 (1.17-1.36) bc	1.25 (1.15-1.36) c	0.95 (0.94- 0.96)
**Preventive**	1.00 a	1.29 (1.17- 1.42) b	1.37 (1.24- 1.52) bc	1.45 (1.28- 1.63) c	1.38 (1.25- 1.52) c	1.40 (1.25-1.57) bc	0.93 (0.92- 0.94)
**Simple Restorative**	1.00 a	1.71 (1.53-1.91) b	1.59(1.41-1.79)b	1.51 (1.31-1.73) bc	1.37 (1.21-1.54) c	1.30 (1.14-1.48) c	1.01 (1.00- 1.03)
**Complex Restorative**	1.00 a	2.13 (1.68- 2.71) b	1.44 (1.10- 1.88) ac	1.29 (0.95- 1.75) ac	1.51 (1.17- 1.96) c	1.39 (1.04- 1.87) ac	1.20 (1.15- 1.25)
**Endodontic**	1.00 a	1.90 (1.53- 2.35) b	1.33 (1.05- 1.68) a	1.26 (0.97- 1.65) a	1.39 (1.11- 1.74) a	1.20 (0.93- 1.55) a	1.14 (1.10- 1.19)
**Extractions**	1.00 a	1.75 (1.53- 2.00) b	1.52 (1.31-1.75) c	1.50 (1.27-1.77) c	1.58 (1.37- 1.81) c	1.51 (1.29-1.77) c	0.99 (0.96-1.03)

^$^ Reference group; ^**+**^ Rate Ratios for change in average number of procedures with a 2-fold (log transformed) increase in ZIP code poverty level across all AHEC regions.

a, b, c, ac, bc- Groups in a row sharing the same letter are not statistically significantly different based on p-values (p < 0.05) adjusted for multiple comparisons using the Tukey-Kramer method.

Table 4 also reports the rate ratios for procedures with an increase in ZIP code poverty level, and results from pairwise comparisons between the AHEC categories. We found that as the ZIP code poverty levels increase, there was a decrease in the number of diagnostic and preventive procedures and an increase in corrective and extraction procedures. In the pairwise comparisons analysis we found that children from Rural 1 and inner-city Milwaukee groups were significantly different from the other AHEC groups for most treatment procedures. For the remaining AHEC categories we found that Rural 2 and Rural 3 were similar to each other, and Urban and suburban Milwaukee groups were similar to each other for most procedures examined.

## Discussion

In this study we examined the patterns of utilization of dental services and procedures among 0-18 year old enrollees in DDWI from 2002 to 2008. The subjects are residents of areas with different levels of urbanization in the state of Wisconsin. To our knowledge this is the first study to compare dental procedures provided to privately insured children living in inner-city and rural areas.

We found that the utilization rates for children from all geographic regions were similar except for those from inner-city Milwaukee, who had much lower rates. These findings are different from previous studies which reported significant differences in utilization rates between rural and urban populations [3,5,8]. In this study, approximately, 67%, 68% and 50% of children from small rural communities, suburban Milwaukee and inner city Milwaukee had at least one annual dental visit, respectively. These rates are lower than the rates reported for rural (69.9%) and urban (73.6%) children by Vargas et al., who analyzed the data from National Health Interview Survey (NHIS) and National Health and Nutrition Examination Survey (NHANES) [5] which are based on representative samples of the United States and include both publicly and privately insured children. They also reported utilization rates of 79.3% and 80.1% for rural and urban children from non-poor families (at or over 200% of the FPL), respectively, [5] which are considerably higher than the rates we found in this privately insured population.

In the analyses comparing receipt of dental procedures, we found that children living in small rural and inner-city areas have fewer preventive and diagnostic procedures. We also found a concomitant increase in use of these procedures and urbanization. Similarly, there was a decrease in the utilization of these procedures with increasing poverty levels in the ZIP codes. The subjects in this study have 100% coverage for diagnostic and preventive procedures, but we still found significant differences in use of these procedures suggesting that the level of urbanization and area poverty play a role in dental access and utilization even in these insured populations.

Compared to children living in other areas, we found that enrollees from inner-city had the lowest, and those from small rural areas had the highest number of restorative and endodontic procedures, respectively. Previous studies reported that rural residents, racial/ethnic minorities and individuals from low socioeconomic backgrounds are more likely to visit a dentist because of a problem or when in pain, necessitating complex restorations, endodontic procedures and extractions [5,12,21,22]. We found that children from Rural 1 regions had fewer visits than children from more urbanized areas, yet they have the highest number of corrective procedures of all groups included in the analysis. Our findings suggest that children in this group may have higher disease levels and/or an episodic pattern of care. On the contrary, children from inner-city region had the fewest procedures of all types when compared to other children. While low overall dental utilization rates for children in this group may explain this finding, it also suggests that they may be facing additional barriers [14] or have higher levels of untreated disease.

Socio-economic status of an individual and his or her place of residence are closely related and have been shown to independently affect the person’s health, access to healthcare and health outcomes [6,23,24]. People living in areas with higher poverty levels have fewer preventive care visits and procedures compared to those who live in high income areas [25-27]. These disparities have been shown to persist even after adjusting for factors such as insurance status, differences in supply of providers, and having regular source of care [25]. The children in this study are enrolled in a private dental insurance plan and are not random representatives of their ZIP codes in terms of poverty. Nonetheless, we found that within each AHEC category, as ZIP code poverty levels increased, there was a decrease in the number of diagnostic and preventive procedures and an increase in endodontic and surgical procedures (data not show). We found a similar pattern from the multivariable analysis showing that ZIP code poverty is an independent predictor of dental care patterns even in this privately insured population.

The utilization rates for all procedures examined in the analysis were lowest among children from inner-city Milwaukee and the poverty levels were among the highest in these ZIP codes. This combination has led to a situation wherein the rate ratios for the treatment procedures from multivariable analysis remain high as opposed to being closer to null after controlling for poverty. This, we suspect, is because of negative confounding. As we projected the low-usage rate of a high poverty group (inner-city Milwaukee) to higher usage rate of groups with lower poverty, the usage of these procedures is expected to be lower than what is actually observed. In other words, since these areas did not have such high poverty levels, the model would expect their rate of usage of these procedures to be much lower than the actual values. This widens the gap between the groups leading to an increase in the magnitude of the rate ratios for these procedures after adjusting for poverty.

### Strengths and limitations

We determined relative rurality or urbanicity of the location of residence using population size living in an urban cluster. While this approach has its drawbacks, it overcomes the limitations of previous methods used for classification. The Rural Urban Commuting Area (RUCA) system, [28] Urban Influence codes [29] and dichotomization into rural and urban areas are among the more commonly used methods for making this classification. The main limitation of the RUCA system is that it is based on commuting patterns which categorizes many non-suburban metro-adjacent areas in the “metropolitan” category. Urban Influence codes are county level measures based on the largest metropolitan area and may misclassify large number of people who live in rural areas within those counties. Using the UIC method will categorize over 30% of the rural populations in the state of Wisconsin as “metropolitan” dwelling [Personal communication from Wisconsin AHEC]. The strategy used by us uses census tract data summarized at the level of place to classify cities, villages and towns at different levels as urban or rural. This strategy would also differentiate large metropolitan areas like Milwaukee from other metropolitan areas, as well as inner-city and suburban Milwaukee areas.

There are limitations to our study that should be noted. First, our dataset did not include child-level information on dental need but only procedure billing codes. Second, our final statistical models did not include factors that have been shown to be associated with patterns of dental care utilization such as availability of providers, parental education and perceptions of need, and family socioeconomic status, however, we employed proxy measures by disaggregating this information from ZIP codes. Our results do seem to suggest that at least shortage of providers is not a significant barrier to care in this population, as we found identical utilizations rates for all groups and the only group with lower rates (inner-city Milwaukee) is close to areas with dental providers. Third, we were unable to examine whether some of the extractions were due to orthodontic treatment, however, it is unlikely that the high rates of extractions in Rural 1 areas would be explained by orthodontic treatments. Finally, our results have limited generalizability because only data for children enrolled in one private dental insurance carrier was analyzed, albeit the largest in the state.

## Conclusions

We found significant geographic variations in dental procedures received by children enrolled in DDWI. Inner-city Milwaukee children had significantly fewer preventive and diagnostic dental procedures and those from the smallest rural communities had higher rates of all other dental procedures.

## Competing interests

None of the authors have any financial or Non-financial competing interests in the project. FE is employed by Delta Dental of Wisconsin but declares no competing interest.

## Authors’ contribution

PB participated in design, acquisition of data, interpretation of the results, and took the lead in writing the manuscript. QX and AS performed the statistical analysis and interpretation of the results. FE helped in acquisition of the data. FE, RK and CO contributed to completion of the manuscript and have critically revised the manuscript. All authors have read and approved the final manuscript will hold themselves jointly and individually responsible for its content.

## Pre-publication history

The pre-publication history for this paper can be accessed here:

http://www.biomedcentral.com/1472-6831/12/58/prepub
